# Upper-Body versus Lower-Body Cooling in Individuals with Paraplegia during Arm-Crank Exercise in the Heat

**DOI:** 10.1249/MSS.0000000000003244

**Published:** 2023-06-30

**Authors:** PUCK ALKEMADE, THIJS M. H. EIJSVOGELS, THOMAS W. J. JANSSEN, KASPAR M. B. JANSEN, BORIS R. M. KINGMA, HEIN A. M. DAANEN

**Affiliations:** 1 Faculty of Behavioral and Movement Sciences, Vrije Universiteit Amsterdam, Amsterdam Movement Sciences, Amsterdam, THE NETHERLANDS; 2 Radboud Institute for Health Sciences, Department of Physiology, Radboud University Medical Center, Nijmegen, THE NETHERLANDS; 3 Amsterdam Institute of Sport Science, Amsterdam, THE NETHERLANDS; 4 Department of Design Engineering, Delft University of Technology, Delft, THE NETHERLANDS; 5 Department Human Performance, Unit Defence, Safety and Security, TNO, The Netherlands Organization for Applied Sciences, Soesterberg, THE NETHERLANDS

**Keywords:** HYPERTHERMIA, THERMOREGULATION, SPINAL CORD INJURY, PARA-ATHLETES, LEG COOLING, PER-COOLING

## Abstract

**Purpose:**

For wheelchair users with a spinal cord injury, the lower body may be a more convenient cooling site than the upper body. However, it remains unknown if leg cooling reduces thermal strain in these individuals. We compared the impact of upper-body versus lower-body cooling on physiological and perceptual outcomes during submaximal arm-crank exercise under heat stress in individuals with paraplegia.

**Methods:**

Twelve male participants with paraplegia (T4–L2, 50% complete lesion) performed a maximal exercise test in temperate conditions, and three heat stress tests (32°C, 40% relative humidity) in which they received upper-body cooling (COOL-UB), lower-body cooling (COOL-LB), or no cooling (CON) in a randomized counterbalanced order. Each heat stress test consisted of four exercise blocks of 15 min at 50% of peak power output, with 3 min of rest in between. Cooling was applied using water-perfused pads, with 14.8-m tubing in both COOL-UB and COOL-LB.

**Results:**

Gastrointestinal temperature was 0.2°C (95% confidence interval (CI), 0.1°C to 0.3°C) lower during exercise in COOL-UB versus CON (37.5°C ± 0.4°C vs 37.7°C ± 0.3°C, *P* = 0.009), with no difference between COOL-LB and CON (*P* = 1.0). Heart rate was lower in both COOL-UB (−7 bpm; 95% CI, −11 to −3 bpm; *P* = 0.01) and COOL-LB (−5 bpm; 95% CI, −9 to −1 bpm; *P* = 0.049) compared with CON. The skin temperature reduction at the cooled skin sites was larger in COOL-LB (−10.8°C ± 1.1°C) than in COOL-UB (−6.7°C ± 1.4°C, *P* < 0.001), which limited the cooling capacity in COOL-LB. Thermal sensation of the cooled skin sites was improved and overall thermal discomfort was lower in COOL-UB (*P* = 0.01 and *P* = 0.04) but not in COOL-LB (*P* = 0.17 and *P* = 0.59) compared with CON.

**Conclusions:**

Upper-body cooling more effectively reduced thermal strain than lower-body cooling in individuals with paraplegia, as it induced greater thermophysiological and perceptual benefits.

Exercise in a hot environment induces hyperthermia, cardiovascular strain, and thermal discomfort, increasing the risk for exertional heat illness and performance decrements (1). These adverse effects of exercise under heat stress may be attenuated through the use of cooling strategies (1–5). In individuals with a spinal cord injury (SCI), thermoregulatory function is likely altered because the lesion may affect both heat production (through reduced active muscle mass) and heat dissipation (through autonomic disruption) (6,7). This may have implications for the use of cooling strategies in individuals with an SCI.

An SCI can damage sensory and motor pathways in the spinal cord, but may also disrupt the autonomic pathways. In case of autonomic disruption, there is reduced afferent input and efferent output to and from the thermoregulatory center in the brain, resulting in impaired sudomotor and vasomotor control below the lesion level (6–8). In addition, impaired sympathetic vasoregulation and loss of motor function below the lesion result in muscle pump inactivity, hampering blood redistribution in the legs (6,9,10). During arm-crank exercise, the inability to redistribute blood below the lesion diminishes the increase in ventricular filling pressure, potentially compromising cardiac output and increasing local heat storage (6,9,10). For individuals with paraplegia (i.e., thoracic, lumbar, or sacral lesion), the magnitude of thermoregulatory impairment depends on the degree of autonomic damage as well as the lesion level, with higher lesions affecting a larger part of the body (6,7,9).

Cooling techniques can be used to reduce thermal strain during exercise in individuals with an SCI (3–5). The majority of studies examined the efficacy of upper-body cooling vests, with contrasting results, whereas, to our knowledge, only one study investigated the effectiveness of lower-body cooling by cooling the feet (3–5,11). Remarkably, to date, it has not been explored whether leg cooling reduces thermal strain during exercise in individuals with an SCI, even though the lower body may be a more convenient cooling site than the upper body as it would not constrain movement during upper-body exercise.

Theoretically, lower-body cooling may have advantages over upper-body cooling in individuals with paraplegia, considering the impaired autonomic control of cutaneous vasomotor and sudomotor responses below the lesion level. In able-bodied individuals, it has been shown that active skin cooling reduces cutaneous blood flow at the cooled skin site (12–14). This likely narrows the temperature difference between the cooling application and the skin, hampering heat exchange. The vasoconstriction response to local cooling is regulated by central and local mechanisms (15), and the absence or impairment of the centrally mediated response in individuals with paraplegia may reduce the undesirable vasoconstriction when cooling the lower body. In addition, when the cooling application covers the skin, for example, when using an ice vest, sweat evaporation from the covered site will be impeded. As the sweating response is absent or low on the lower body of individuals with paraplegia, lower-body cooling may only have a limited effect on the body’s evaporative heat loss, while still allowing sweat evaporation on the unaffected upper body.

On the other hand, the effectiveness of lower-body cooling may be restricted by the inactivity of the leg muscles. The lack of local heat production and inactivity of the skeletal muscle pump likely diminish the amount of heat transferred from the muscles to the skin through conduction and arterial blood flow. Such limited heat supply from deeper tissue to the skin likely reduces the temperature difference between the cooling application and the skin and therefore the potential for heat exchange. In addition, the impaired venous return may hamper redistribution of the cooled blood to the upper body, potentially limiting the effect of cooling on body core temperature and further reducing the cooling potential. In line with this theory, Hagobian et al. (11) found that in individuals with an SCI, foot cooling only lowered tympanic temperature during exercise in the heat when blood return from the lower limbs was enhanced using a negative pressure device (alternating negative and neutral pressure, simulating the skeletal muscle pump). Foot cooling without negative pressure did not reduce tympanic temperature, suggesting that the inactive skeletal muscle pump limits the effectiveness of lower-body cooling. However, further research into lower-body cooling is warranted considering their small sample size (*n* = 6) and the relatively small surface area that was cooled (i.e., only the feet). In addition, in individuals with paraplegia, lower-body cooling may have less perceptual benefit compared with upper-body cooling because of absent or disturbed sensory function below the lesion (6,7).

The aim of this study was to compare the impact of upper-body versus lower-body cooling on physiological and perceptual outcomes during submaximal arm-crank exercise under heat stress in individuals with paraplegia. For this purpose, individuals with paraplegia performed three exercise heat stress tests on separate occasions with no cooling, upper-body cooling, and lower-body cooling in a randomized counterbalanced order. For the application of upper-body and lower-body cooling, we used water-perfused pads, with the aim to cover the unaffected portion of the torso and affected portion of the legs, respectively.

## METHODS

### Participants

An *a priori* power analysis was performed in G*Power 3 (16) for peak gastrointestinal temperature (*T*_gi_). To detect an effect of 0.3°C between a cooling condition and the control condition (corresponding to Cohen’s *f* = 0.35), a minimal sample size of 10 was required (for correlations among repeated measures = 0.7, *α* error probability = 0.05, power = 0.8, and nonsphericity correction = 1).

Twelve male participants with paraplegia volunteered to participate in this study (Table 1). We included individuals with an SCI between T4 and L2 (i.e., paraplegia) to ensure there was sufficient unaffected body surface area above the lesion for upper-body cooling, as well as sufficient affected body surface area below the lesion for lower-body cooling. Only male participants were included to facilitate tight contact between the skin and cooling pad at the chest. Participants did not reside in a warm environment (>25°C air temperature) for longer than 5 d within the month before the study. None had been previously diagnosed with exertional heat illness. Procedures were approved by the Ethics Committee of the Faculty of Behavioral and Movement Sciences of the Vrije Universiteit Amsterdam (VCWE-2020-015) and conform to the standards set out by the Declaration of Helsinki. Before participation, participants were informed about the procedures and provided verbal and written consent.

**TABLE 1:** Participant characteristics (*n* = 12).

Characteristic	
Age (yr)	49 ± 12
Height (cm)	182.5 ± 10.2
Body mass (kg)	81.6 ± 12.3
Self-reported weekly training volume (h)	10 ± 6
V̇O_2peak,kg_ (mL⋅kg^−1^⋅min^−1^)	28.8 ± 6.7
Peak power output (W)	154 ± 31
Lesion level	T4–T5, *n* = 3; T6–T12, *n* = 8; L2, *n* = 1
Lesion completeness	AIS A (complete), *n* = 6; AIS C (incomplete), *n* = 3; AIS D (incomplete), *n* = 3
Time since injury (yr)	9 (8–25)

Descriptive statistics are presented as mean ± SD or median (interquartile range).

AIS, The American Spinal Injury Association Impairment Scale; V̇O_2peak,kg_, peak oxygen uptake relative to body mass.

### Study Design

Participants visited the laboratory four times, with 7 (7–14) (median (interquartile range)) d between visits. They were instructed to avoid strenuous exercise in the 24 h preceding each visit. During the first visit, participants completed a graded exercise test in temperate conditions to determine peak oxygen uptake and peak power output. During the three subsequent visits, participants completed a heat stress test in which we applied, in a randomized counterbalanced order, upper-body cooling (COOL-UB), lower-body cooling (COOL-LB), or no cooling (CON). Participants wore shorts and a tight sports base layer shirt in all conditions. For each participant, all heat stress tests were completed on the same time of the day. Exercise was performed on an asynchronous arm-crank ergometer (Angio; Lode B.V., Groningen, the Netherlands) in a climate chamber (b-Cat B.V., Tiel, the Netherlands). Testing took place outside of summer months (October–May; the Netherlands).

### Graded Exercise Test

Participants completed a graded exercise test in temperate conditions (20°C, 40% relative humidity). They started cycling at 10 W for 3 min, after which power output increased with 10 W·min^−1^ until volitional exhaustion. During exercise, strong verbal encouragement was given. The rate of oxygen consumption was monitored breath-by-breath using a metabolic cart (Quark CPET; COSMED, Rome, Italy). Values were discarded if they exceeded 2 SD from the mean within a local 12-s window. Peak oxygen uptake was defined as the highest 15-s moving average. Peak power output was calculated as: peak power output (W) = workload in last complete step (W) + ((time in last incomplete step (min) ÷ step duration (min)) × step size (W)).

### Heat Stress Tests

#### Testing procedure

Upon arrival at the laboratory, participants provided a urine sample, from which urine-specific gravity was measured using a handheld refractometer (PAL-10S; Atago Co. Ltd, Tokyo, Japan). A urine-specific gravity value of ≤1.025 was considered as an indication of sufficient hydration (17). This value was exceeded in one participant on two occasions; after consuming ~5 mL·kg^−1^ of water, he was allowed to resume the experiment. After instrumentation, participants entered the climate chamber (32.4°C (32.3°C–32.5°C), 40% (39%–40%) relative humidity, and minimal airflow), where they first rested for 30 min. Baseline values were obtained during minutes 10 to 15. In COOL-LB and COOL-UB trials, cooling was activated after 15 min of rest and remained activated throughout the whole trial. After the 30-min resting period, arm cranking commenced with a 5-min warm-up at 20 W, followed by four exercise blocks of 15 min at 50% of peak power output. Between exercise blocks, participants rested for 3 min. After the last exercise block, participants were monitored during 15 min of recovery. Participants were not allowed to drink during the heat stress tests. A graphical overview of the heat stress test protocol is presented in Supplemental Figure 1, Supplemental Digital Content 1, http://links.lww.com/MSS/C881.

#### Measurements

*T*_gi_ was measured at 30-s intervals using a validated telemetric capsule (e-Celcius Performance; BodyCap, Caen, France) (18,19), which participants ingested 8 (7–10) h before each heat stress test. Skin temperature (*T*_sk_) was measured on the forehead, and underneath the cooling pads on the mid-upper back, mid-chest, left thigh, and left calf at 1-min intervals using wireless temperature sensors (DS1922; Maxim Integrated Products, Inc., San Jose, CA), attached to the skin with tape (Fixomull Stretch ADH; BSN Medical GmbH, Hamburg, Germany). The *T*_sk_ sensors covered by the cooling pads were placed underneath the center of the pads, where no tubing was present. The mean of chest and upper back *T*_sk_ was used as a measure of upper-body *T*_sk_ (representing skin surface underneath upper-body cooling pads), whereas the mean of thigh and calf *T*_sk_ was used as a measure of lower-body *T*_sk_ (representing skin surface underneath lower-body cooling pads). Heart rate was measured continuously using a chest-belt (Polar Vantage-M, Kempele, Finland).

Local sweat rates (LSR) on the forehead, right scapula, and right thigh were measured continuously using an in-house validated ventilated sweat capsule system (20–22). Forehead LSR was measured during all heat stress tests, whereas scapula LSR was measured only during CON and COOL-LB, and thigh LSR only during CON and COOL-UB. Each capsule had an inner surface area of 3.8 cm^2^ and was applied to the skin using double-sided tape (3M™ Medical Tape 1522; 3M, Saint Paul, MN) and medical glue (Collodion; Mavidon, Flat Rock, NC). Dry nitrogen gas was supplied to the inlet of the capsules, with constant flow rates of 725, 580, and 207 mL·min^−1^ for the forehead, scapula, and thigh, respectively (flowmeter; Omega Engineering, Stanford, CT). These local flow rates were determined based on the expected sweat rate (i.e., higher sweat rate requires higher nitrogen flow rate). Relative humidity of the effluent air was measured ~1 m downstream of the capsule (HygroVUE10; Campbell Scientific, Logan, UT). LSR was calculated as (21):


$$\mathrm{LSR}=\left(\frac{\mathrm{RH}}{100}\right)\cdot \rho \cdot \text{flow rate}$$ [1]

with LSR in kg·s^−1^, RH is the relative humidity downstream of the capsule in %, *ρ* is the nitrogen density at saturated vapor pressure at a given temperature (calculated based on the equation of Antoine (1888) and the ideal gas law) in kg·m^−3^, and nitrogen flow rate in m^3^·s^−1^.

Thermal sensation, thermal discomfort, and rating of perceived exertion were collected in the last minute of each 15-min (resting) and exercise block. Whole-body thermal sensation and local thermal sensation for the chest, back, thigh, and calf were rated on a 9-point scale ranging from −4 (very cold) to +4 (very hot) (23). The mean of chest and back thermal sensation was used as a measure of upper-body thermal sensation (representing sensation underneath upper-body cooling pads), whereas the mean of thigh and calf thermal sensation was used as a measure of lower-body thermal sensation (representing sensation underneath lower-body cooling pads). Thermal discomfort was rated on a 5-point scale ranging from 0 (comfortable) to 4 (extremely uncomfortable) (23). Rating of perceived exertion was rated on a 15-point scale ranging from 6 (very, very light) to 20 (maximal exertion) (24).

Climate chamber conditions were measured using a humidity and temperature probe (HMP9; Vaisala, Vantaa, Finland). Body mass (wearing shorts) was measured before and immediately after each heat stress test to estimate whole-body sweat loss. Eight participants were weighed in their wheelchair to the nearest 100 g (BL-5R; Christen Swiss B.V., Geldermalsen, the Netherlands), after which wheelchair mass (measured preexercise) was subtracted to calculate body mass. Four participants were weighed in standing position to the nearest 5 g (SATEX 34 SA-1 250; Weegtechniek Holland B.V., Zeewolde, the Netherlands).

#### Cooling strategy

**FIGURE 1:**
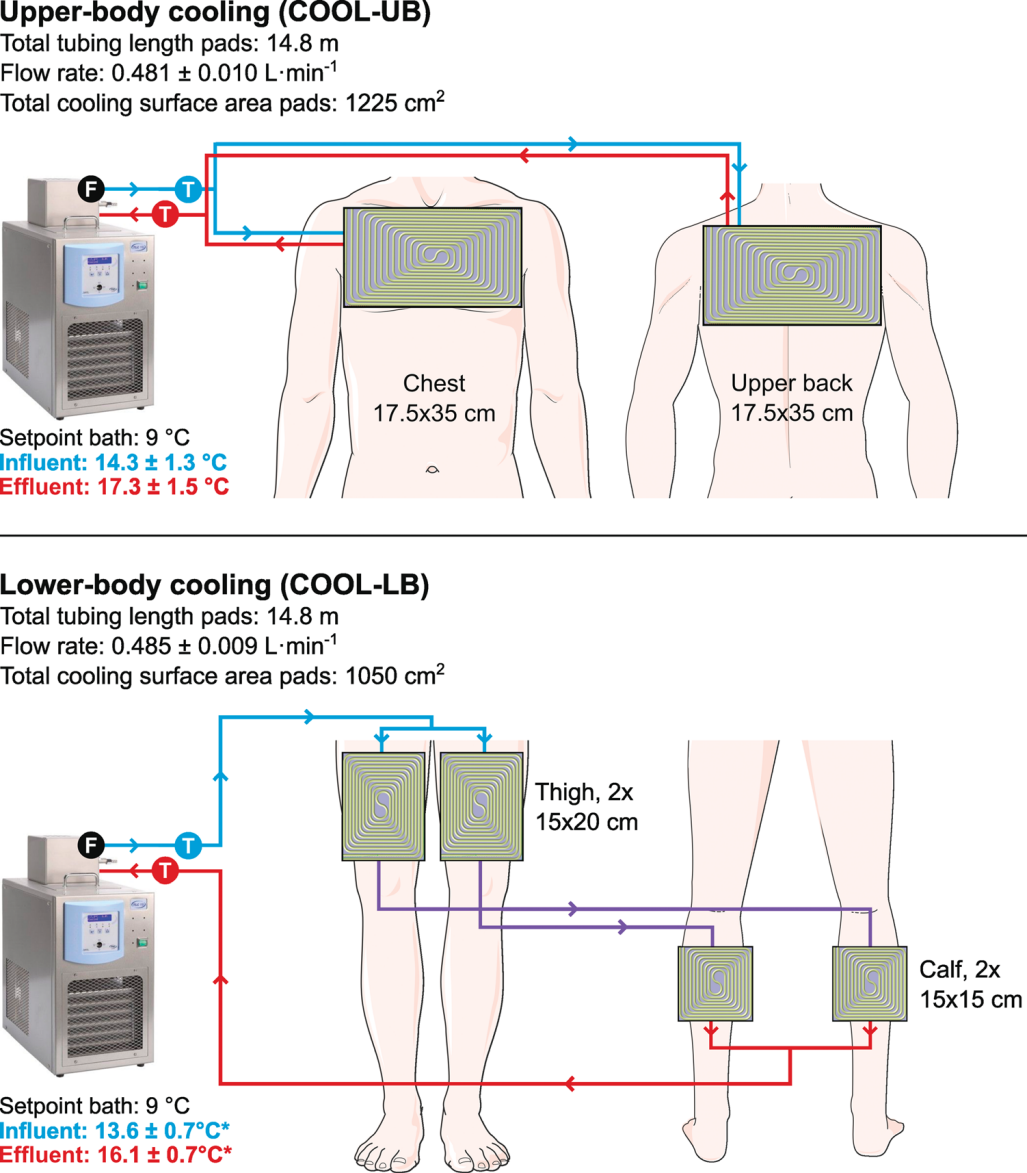
Schematic overview of the cooling systems in COOL-UB and COOL-LB. *Blue* and *red lines* represent effluent and influent water to the pads, respectively. “F” and “T” represent the flow rate and temperature measurements of circulating water, respectively. *Influent and effluent water temperatures were lower in COOL-LB than in COOL-UB (*P* < 0.001).

Cooling pads consisted of flexible tubing (Tygon S3™ B-44-3; inner diameter, 3.18 mm; outer diameter, 4.76 mm) sewn to a layer of net fabric, with the tubing being in direct contact with the skin. In COOL-UB, two cooling pads were applied to the skin of the chest and upper back, with the aim to cover the unaffected area of the torso (Fig. 1). In COOL-LB, four cooling pads were applied to the skin of the thighs and calves, with the aim to cover the affected portion of the lower body. To ensure an equal contact area between the pads and the skin in both cooling conditions, we standardized total tubing length of the pads. Because of the different dimensions of the upper-body and lower-body pads, this resulted in a larger total surface area for the pads in COOL-UB and higher tubing density (i.e., tubing length/surface area) for the pads in COOL-LB (COOL-LB vs COOL-UB, 1.4 vs 1.2 cm·cm^−2^). The tubing on the cooling pads was connected to a thermostat bath (TLC15-5; Tamson Instruments B.V., Bleiswijk, the Netherlands). The temperature of influent and effluent water was measured ~1 m upstream and downstream of the cooling pads using thermocouples (Type K, RS PRO; RS Components B.V., Corby, UK) inserted through the tubing. Water flow rate was similar in both conditions (measured using flowmeter; FCH-midi-POM; B.I.O.-TECH e.K., Vilshofen, Germany). In COOL-UB, cooling pads were placed underneath the tight base layer shirt (honeycomb fabric; 17% spandex, 83% polyester cationic), which was used to improve the contact between skin and cooling pads. In the COOL-LB conditions, cooling pads were applied to the skin using tubular bandage (Tubigrip; Mölnlycke Health Care, Gothenburg, Sweden), ensuring good skin–pad contact.

### Cooling Capacity

We performed hotplate experiments to assess the cooling capacity of the water-perfused pads. The custom-made guarded hotplate aims to keep the preset temperature of the aluminum surface constant by adjusting the power input to the plate (25). When a cooling pad is placed on the hotplate, the power required to keep the preset temperature is a measure of the cooling capacity (i.e., the amount of heat that the cooling pad extracts from the hotplate). We expected the cooling capacity per unit surface area (*C*_cool/SA_; W·m^−2^) to be proportional to the difference between the temperature of the water flowing through the cooling pad (*T*_water_; °C) and the temperature of the hotplate (*T*_hotplate_; °C):


$$C_{\text{cool}/\mathrm{SA}}=a\times \left(T_{\text{hotplate}}-T_{\text{water}}\right)+b$$ [2]

 Parameters *a* and *b* primarily depend on the environmental conditions, the absolute values of *T*_hotplate_ and *T*_water_, and the water flow rate. To obtain a linear fit representative of our heat stress test, we placed the hotplate setup in a bench-top climatic chamber (Espec SH-661; Espec Corp., Osaka, Japan) set to 32°C and 40% relative humidity, and wrapped a tubular bandage around the cooling pad and hotplate to ensure optimal contact. Cooling was applied to the hotplate using the calf cooling pad, which had the same size as the measurement area of the hotplate (15 × 15 cm). We measured cooling capacity at four hotplate temperatures of 25°C, 29°C, 33°C, and 37°C (matching *T*_sk_ heat stress tests) and two water temperatures of 14.5°C and 18.7°C (matching influent/effluent water temperatures heat stress tests). Water flow rate was set to ~0.25 L·min^−1^ to match the flow rate during the heat stress tests (where pads were connected in parallel, i.e., flow rate ÷ 2). We used these data to determine *a* and *b* in equation 2, and subsequently used *a* and *b* to estimate the cooling capacity of the lower-body and upper-body cooling systems during the heat stress tests (*C*_cool_; W), using:


$$C_{\text{cool}}=\left(a\times \left(T_{\mathrm{sk}}-T_{\text{water}}\right)+b\right)\times \mathrm{SA}\;\left(m^{2}\right)$$ [3]

where *T*_sk_ is the temperature of the cooled skin site (°C), and *T*_water_ is the temperature of the water flowing through the pads (°C), both measured during COOL-UB or COOL-LB. SA is the total surface area of the cooling pads in COOL-UB or COOL-LB.

### Data Analysis

All data were synchronized and formatted using MATLAB (R2021b; The MathWorks Inc., Natick, MA). Further (statistical) analyses were performed using R software (version 4.1.1; R Foundation for Statistical Computing, Vienna, Austria) in the Rstudio environment (version 2021.09.0+351; Rstudio, Inc., Boston, MA). The level of statistical significance was set at *P* < 0.05. Data were reported as mean ± SD in case of normal distribution or as median (interquartile range) in case of nonnormal distribution and for categorical variables. Mean or median differences (MD) were reported with a 95% confidence interval (CI) or first and third quartile (interquartile range), respectively.

To investigate the impact of cooling site on physiological variables during exercise, a two-way repeated-measures ANOVA was performed, with type of cooling (CON, COOL-UB, and COOL-LB) and time (four exercise blocks) as within-participant factors. Both the Shapiro–Wilk test and visual inspection were used to assess normality of the variable in each cell of the design. We used the Greenhouse–Geisser epsilon to assess sphericity: if epsilon was ≥0.75, the Huynh–Feldt correction was applied, and for epsilon <0.75, the Greenhouse–Geisser correction was applied (26). In case of a significant main effect across cooling conditions, with no significant interaction effect (condition–time), paired *t*-tests with Bonferroni correction were used to identify differences between cooling condition means. In case of a significant interaction effect, a one-way repeated-measures ANOVA, with condition as within-participant factor, was performed for all four exercise blocks. If the cooling condition effect was significant, paired *t*-tests with Bonferroni correction were used to identify differences between cooling conditions in that exercise block.

To investigate the influence of cooling condition on peak values for *T*_gi_ and heart rate (i.e., highest value measured), ∆*T*_gi_ (i.e., peak minus baseline), whole-body sweat loss, and median perceptual scores, a one-way repeated-measures ANOVA (in case of normal distribution) or Friedman test (in case of nonnormal distribution, and categorical variables) was performed. In case of a significant effect, pairwise *t*-tests or Wilcoxon signed rank tests with Bonferroni correction were used to identify differences between conditions. *P* values derived from nonparametric tests (Friedman, Wilcoxon signed rank) were reported with subscript “np.” An overview of all test statistics, *P* values, and (un)standardized effect sizes is provided in Appendix 1, Supplemental Digital Content 2, http://links.lww.com/MSS/C882.

## RESULTS

Average external power output during the heat stress tests was 75 (68–83) W. For two participants, power output was reduced during the first heat stress test, as they were unable to sustain the target power output. The adjusted protocol was replicated in the subsequent heat stress tests. On average, they both cycled on a power output corresponding to 43% of their peak power output (instead of the targeted 50%). For the analysis of heart rate, data of one participant were removed as he had a low heart rate due to medical reasons causing extreme outlier data (during exercise 50 bpm below average). Hence, the analytical data set for heart rate included *n* = 11. Because of missing data, the analytical data set included *n* = 11 for forehead *T*_sk_, upper-body *T*_sk_, lower-body *T*_sk_, and forehead LSR, and *n* = 10 for whole-body sweat loss.

### Gastrointestinal temperature

**FIGURE 2:**
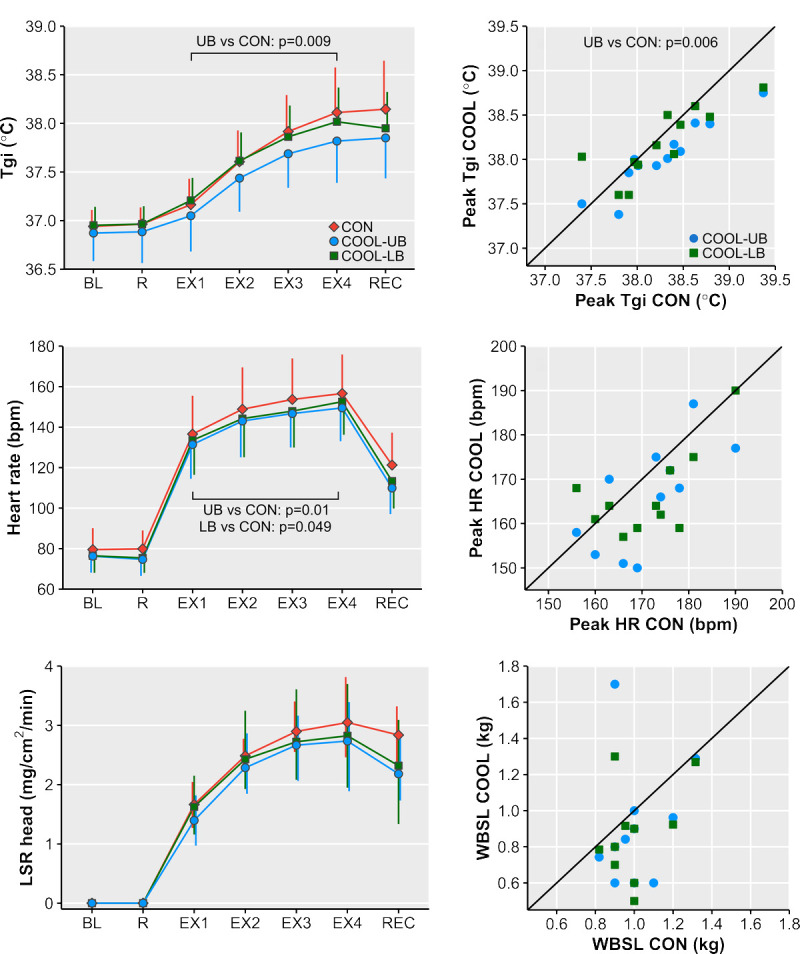
*Left*: *T*_gi_ (A; *T*_gi_; mean ± SD), heart rate (C; mean ± SD), and LSR on the forehead (E; LSR head; median (Q1–Q3)) over time in the control (CON), upper-body cooling (COOL-UB), and lower-body cooling (COOL-LB) conditions. *Right*: Scatterplots of individual data points with identity line for peak *T*_gi_ (B), peak heart rate (D), and whole-body sweat loss (F; WBSL), with values for CON on the *x* axis and values for both cooling (COOL) conditions on the *y* axis. When a data point falls below the identity line, the value in COOL is lower than in CON. Only *P* values <0.05 are displayed. BL, baseline; EX, 15-min exercise block; R, 15-min rest; REC, 15-min recovery.

*T*_gi_ (*n* = 12) was different across cooling conditions (*P* = 0.03), and this difference tended to change over time (condition–time, *P* = 0.051; Fig. 2A). Mean *T*_gi_ was lower in COOL-UB than CON (MD (CI), −0.2°C (−0.3°C to −0.1°C); *P* = 0.009), whereas there was no difference between COOL-LB and CON (*P* = 1.0). Explorative analysis revealed that *T*_gi_ was lower in COOL-UB compared with CON in exercise block 3 (MD (CI), −0.2°C (−0.3°C to −0.1°C); *P* = 0.002) and exercise block 4 (MD (CI), −0.3°C (−0.4°C to −0.2°C); *P* = 0.002).

Peak *T*_gi_ (*n* = 12) was different across cooling conditions (*P* = 0.01), with peak *T*_gi_ being lower in COOL-UB versus CON (MD (CI), −0.2°C (−0.4°C to −0.1°C); *P* = 0.006), but comparable in COOL-LB versus CON (*P* = 0.88; Fig. 2B). When peak *T*_gi_ was expressed relative to baseline (i.e., ∆*T*_gi_; *n* = 12), there was initially no difference across cooling conditions (*P* = 0.26), but this finding was strongly influenced by abnormal *T*_gi_ data at rest in one participant (see Appendix 2, Supplemental Digital Content 3, http://links.lww.com/MSS/C883). Secondary analyses excluding the respective participant confirmed our that main findings as peak *T*_gi_ (*n* = 11) was different across cooling conditions (*P* < 0.001), with peak *T*_gi_ being lower in COOL-UB versus CON (MD (CI), −0.3°C (−0.4°C to −0.1°C); *P* = 0.002). Similarly, ∆*T*_gi_ (*n* = 11) was different across cooling conditions (*P* = 0.02), with ∆*T*_gi_ being lower in COOL-UB versus CON (MD (CI), −0.3°C (−0.4°C to −0.1°C); *P* = 0.02).

### Heart rate

Heart rate (*n* = 11) was different across cooling conditions (*P* = 0.004), with the difference being constant over time (condition–time *P* = 0.52; Fig. 2C). Mean heart rate was lower in both COOL-UB (MD (CI), −7 (−11 to −3) bpm; *P* = 0.01) and COOL-LB (MD (CI), −5 (−9 to −1) bpm; *P* = 0.049) compared with CON. Peak heart rate was similar across cooling conditions (*P* = 0.10; Fig. 2D).

### Skin temperatures

**FIGURE 3:**
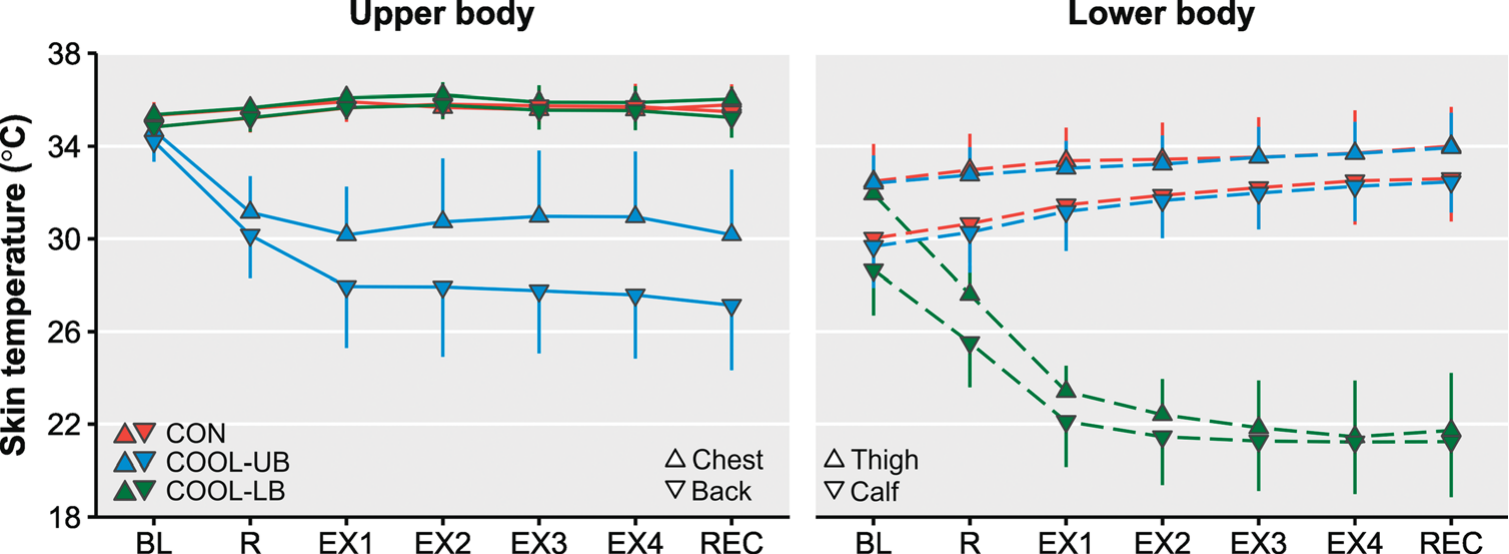
*T*_sk_ on the upper and lower body (mean ± SD) over time in the control (CON), upper-body cooling (COOL-UB), and lower-body cooling (COOL-LB) conditions. BL, baseline; EX, 15-min exercise block; R, 15-min rest; REC, 15-min recovery.

Forehead *T*_sk_ (*n* = 11) was similar across cooling conditions (*P* = 0.36). Upper-body *T*_sk_ (*n* = 11) was different across cooling conditions (*P* < 0.001), with the difference being constant over time (condition–time *P* = 0.35; Fig. 3). Mean upper-body *T*_sk_ was lower in COOL-UB compared with both CON (MD (CI), −6.7°C (−7.7 to −5.8°C)°C; *P* < 0.001) and COOL-LB (MD (CI), −6.8°C (−7.9 to −5.7°C); *P* < 0.001). Lower-body *T*_sk_ (*n* = 11) was different across cooling conditions (*P* < 0.001), with the difference changing over time (condition–time *P* < 0.001; Fig. 3). In all four exercise blocks, lower-body *T*_sk_ was lower in COOL-LB compared with both CON (MD, −11.7°C to −9.7°C; all *P* < 0.001) and COOL-UB (MD, −11.7°C to −9.4°C; all *P* < 0.001). The *T*_sk_ reduction at the cooled skin sites was larger in COOL-LB (−10.8°C ± 1.1°C) than in COOL-UB (−6.7°C ± 1.4°C, *P* < 0.001).

### Local and whole-body sweat rates

Forehead LSR (*n* = 11) was similar across cooling conditions (*P* = 0.30; Fig. 2E). Whole-body sweat loss (*n* = 10) was different across cooling conditions (*P*_np_ = 0.01), but pairwise comparisons could not reveal differences between conditions (COOL-UB vs CON: *P*_np_ = 0.37, COOL-LB vs CON: *P*_np_ = 0.20, COOL-UB vs COOL-LB: *P*_np_ = 1.0; Fig. 2F). Mean LSR on the scapula (*n* = 12) during exercise was not different between COOL-LB and CON (1.2 ± 0.6 vs 1.2 ± 0.5 mg·cm^−2^·min^−1^; *P* = 0.61). On the thigh (*n* = 12), no sweat rate was detected in four participants (SCI; American Spinal Injury Association Impairment Scale (AIS) A, T4–T7). For the other eight participants (SCI; 2× AIS A, 6× AIS C/D, T4–L2), mean LSR on the thigh during exercise was not different between COOL-UB and CON (0.2 (0.0–0.6) vs 0.3 (0.0–0.5) mg·cm^−2^·min^−1^; *P* = 0.18).

### Perceptual variables

**FIGURE 4:**
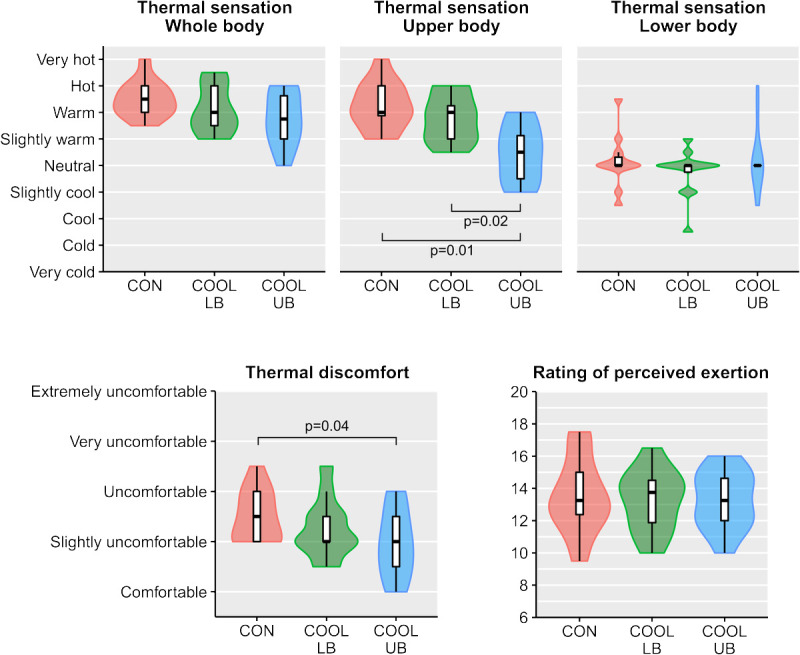
Violin plots with boxplot overlay for median perceptual scores during exercise, in the control (CON), lower-body cooling (COOL-LB), and upper-body cooling (COOL-UB) conditions.

Median whole-body thermal sensation was similar across cooling conditions (*P*_np_ = 0.07; Fig. 4). Median upper-body thermal sensation was different across cooling conditions (*P*_np_ < 0.001), with lower values in COOL-UB compared with both CON (MD (Q1–Q3), −1.8 (−2.6 to −1.0); *P*_np_ = 0.01) and COOL-LB (MD (Q1–Q3), −1.3 (2.0 to −0.5); *P*_np_ = 0.02). Median lower-body thermal sensation was different across cooling conditions (*P*_np_ = 0.02), but pairwise comparisons could not reveal differences between conditions (COOL-UB vs CON: *P*_np_ = 1.0, COOL-LB vs CON: *P*_np_ = 0.17, COOL-UB vs COOL-LB: *P*_np_ = 0.27). Median thermal discomfort was different across cooling conditions (*P*_np_ = 0.02; Fig. 4), with lower values in COOL-UB than CON (MD (Q1–Q3), −0.5 (−1.0 to −0.4); *P*_np_ = 0.04), but with similar values for COOL-LB and CON (*P*_np_ = 0.59). Median rating of perceived exertion was similar across cooling conditions (*P*_np_ = 0.98; Fig. 4).

### Cooling capacity

**FIGURE 5:**
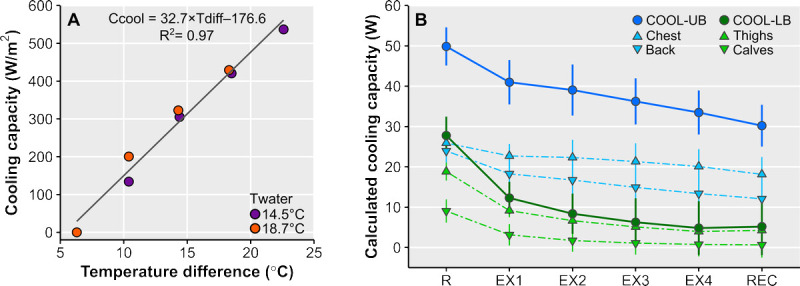
A, Cooling capacity (*C*_cool_) as a function of the temperature difference (*T*_diff_) between the hotplate and influent water (*T*_water_). B, Cooling capacity of the cooling pads over time, calculated according to equation 3, in the upper-body cooling (COOL-UB) and lower-body cooling (COOL-LB) conditions. Cooling capacity of the chest pad plus upper-back pad equals COOL-UB cooling capacity. Cooling capacity of the two calf pads plus two thigh pads equals COOL-LB cooling capacity. EX, 15-min exercise block; R, 15-min rest; REC, 15-min recovery.

A strong linear relationship was found between cooling capacity and the temperature difference between the hotplate and water (Fig. 5A). Using the fitted linear model, we calculated the cooling capacity during COOL-UB and COOL-LB (Fig. 5B).

## DISCUSSION

In this study, we compared the impact of upper-body versus lower-body cooling on physiological and perceptual outcomes during submaximal arm-crank exercise under heat stress in individuals with paraplegia. We found that upper-body cooling attenuated the exercise-induced rise in *T*_gi_ and heart rate, whereas lower-body cooling only reduced heart rate. Furthermore, upper-body cooling provided a perceptual benefit, with participants reporting a less warm sensation of the upper body and an overall lower thermal discomfort. Lower-body cooling did not alter lower-body thermal sensation, suggesting that participants did not perceive the cooling. These findings indicate that, in individuals with paraplegia, upper-body cooling is more effective in lowering thermal strain and improving thermal perception compared with lower-body cooling during exercise in the heat.

We observed that upper-body cooling attenuated the exercise-induced rise in *T*_gi_, whereas lower-body cooling did not. The absence of a *T*_gi_ decrease in COOL-LB may primarily be explained by the inactivity of the leg muscles. The limited local heat production and the skeletal muscle pump inactivity likely diminished transport of warm blood from the muscles to the skin, hampering heat transfer from the skin to the cooling pad. In addition, the inactive muscle pump (in combination with impaired sympathetic control) in individuals with paraplegia likely attenuates the rise in venous return, which may have caused partial retention of cooled blood in the legs (9–11). These suggestions are supported by our *T*_sk_ data, as lower-body cooling resulted in a larger temperature reduction at the cooled skin site compared with upper-body cooling. We also showed that this large *T*_sk_ reduction in COOL-LB reduced the temperature difference between the cooling pad and the skin, limiting cooling capacity. In contrast, in COOL-UB, the exercising upper body muscles induced local heat production and stimulated blood flow resulting in higher *T*_sk_, enhancing heat extraction by the cooling pad.

These findings contradict our hypothesis in favor of lower-body cooling. We proposed that heat exchange between the cooling pad and the skin would be improved when the vasoconstriction response is attenuated. We did not measure skin blood flow during exercise, but it seems plausible that the aggressive skin cooling induced cutaneous vasoconstriction during COOL-LB, even in case of sympathetic dysfunction, as this response is not only centrally but also locally mediated (12,15). Furthermore, active vasodilator activity in response to hyperthermia may inhibit the vasoconstriction response to local cooling (27). This inhibitory effect may be absent below the lesion when sympathetic vasoregulation is impaired, which could, in contrast to our hypothesis, result in an amplified vasoconstriction response during lower-body cooling. We also proposed that heat loss through sweat evaporation may be less affected when cooling the lower body, given the absent or low sweat rate below the lesion level. However, this potential advantage of lower-body cooling may be outweighed by the limited blood redistribution in the lower body and the consequent reduction in cooling capacity.

Interestingly, lower-body cooling attenuated the exercise-induced increase in heart rate, in the absence of a *T*_gi_ reduction. This may be explained by the (locally mediated) cutaneous vasoconstriction response to lower-body cooling, which may have led to an increased central blood volume, enabling a greater ventricular filling and consequently a larger stroke volume, allowing a lower heart rate to maintain cardiac output in COOL-LB relative to CON (1). Such reduction in cardiovascular strain has the potential to attenuate performance decrements during exercise in the heat (1,28). Thus, even though lower-body cooling does not attenuate the rise in *T*_gi_, it may lower cardiovascular exercise demands and induce performance benefits.

Our participants clearly perceived the cooling during COOL-UB. During COOL-LB, the cooling perception was highly variable, with no overall benefit relative to CON. The absent perception of cooling in COOL-LB was most likely the result of lacking or disturbed sensory feedback from the insensate lower body to the brain (6). A cooler thermal perception and lower thermal discomfort can enhance motivation and willingness to continue exercising in the heat, and may therefore positively influence exercise capacity (1,29). Lower-body cooling may induce these perceptual benefits in individuals with intact sensory function below the lesion. However, in individuals with disturbed sensation in the legs, upper-body cooling provides more perceptual benefit than lower-body cooling.

### Strengths and limitations

This study was the first to compare the effectiveness of upper-body and lower-body cooling in individuals with an SCI. Our research provides fundamental insights into the effectiveness of cooling in individuals with paraplegia and may give direction to cooling use in practice as well as to future cooling studies. Even though this study was performed within a well-controlled laboratory setting, some limitations should be acknowledged. First, the total surface area of the cooling pads was larger in COOL-UB than in COOL-LB. However, the pads in COOL-LB had a higher tubing density than those in COOL-UB, resulting in an equal contact area between pads and skin across cooling conditions. We therefore think that the different surface area did not influence the applied cooling dose. Second, LSR on the scapula and thigh was measured only on the right side of the body. Local differences in sweat rate may exist, diminishing the generalizability of these measurements.

### Practical implications

We observed that upper-body cooling reduced peak *T*_gi_ with 0.2°C. This reduction may appear small but was observed relative to an exercise-induced *T*_gi_ rise of 1.3°C, that is, a 17% reduction. Under more demanding conditions, in which the exercise-induced rise in core temperature is likely larger, cooling may induce greater core temperature reductions than observed in this study (11,30). In addition, the *T*_gi_ difference between COOL-UB and CON tended to increase toward the end of exercise, suggesting that the effect of upper-body cooling on *T*_gi_ may be larger for exercise of longer duration. Furthermore, in our study, upper-body cooling pads only covered the upper torso, aiming to cool the unaffected portion of the upper body. In practice, a cooling vest may cover the entire torso, which potentially enhances the magnitude of cooling benefits. This may, however, not hold for individuals with a high-level SCI, as it has been observed that individuals with a high thoracic SCI benefited less from torso cooling than those with a mid to low thoracic SCI (31).

In this study, we assessed the effectiveness of cooling during exercise, as the difference between upper-body and lower-body cooling may be more pronounced during exercise than in rest. In a real-world sports setting, wearing a cooling vest during competition may be impractical or against the regulations, and in those cases, cooling before exercise (i.e., precooling) may be preferred. It has been shown that upper-body precooling has the potential to effectively reduce thermal strain during subsequent exercise in individuals with an SCI (32–34). To the best of our knowledge, lower-body precooling has not yet been investigated but may, like lower-body per-cooling, not attenuate thermal strain considering the limited local heat production and blood redistribution in the legs.

Altogether, our findings suggest that upper-body cooling is preferred over lower-body cooling during exercise in individuals with paraplegia. Recently, we showed that the use of cooling vests accounted for respectively 8% and 15% of all precooling and per-cooling strategies used by Paralympic athletes competing at the Tokyo 2020 Games (35). This relative unpopularity of cooling vests may be related to their weight and the uncomfortable fit for individuals exercising in a wheelchair or hand bike. To improve the practicality of cooling vests for this group, their fit may need to be customized to the individual user.

## CONCLUSIONS

In individuals with paraplegia, upper-body cooling attenuated increases in *T*_gi_ and heart rate, and lowered thermal discomfort during submaximal arm-crank exercise in the heat, whereas lower-body cooling only reduced heart rate. Thus, when applying a cooling strategy in individuals with paraplegia during upper-body exercise in the heat, upper-body cooling is preferred over lower-body cooling.

## Supplementary Material
