# Supplementary material for: Upper-Body versus Lower-Body Cooling in Individuals with Paraplegia during Arm-Crank Exercise in the Heat
Source: Med Sci Sports Exerc. 2023 Jun 30;55(11):2014–24. doi: 10.1249/MSS.0000000000003244 (PMC10581408; doi:10.1249/MSS.0000000000003244)
Supplement: Supplementary file 1 [file msse-55-2014-s001.pdf]

Supplemental Digital Content 1

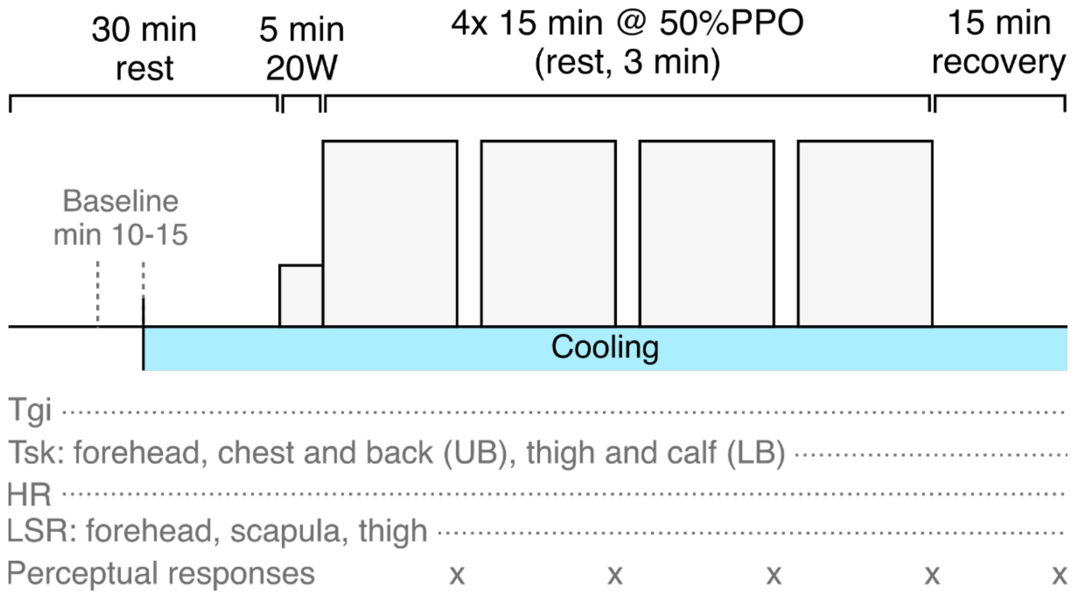

Supplemental Figure 1. Heat stress test protocol and measurements. PPO, peak power output; Tgi, gastrointestinal temperature; Tsk, skin temperature; HR, heart rate; LSR, local sweat rate.
