# Supplementary material for: Upper-Body versus Lower-Body Cooling in Individuals with Paraplegia during Arm-Crank Exercise in the Heat
Source: Med Sci Sports Exerc. 2023 Jun 30;55(11):2014–24. doi: 10.1249/MSS.0000000000003244 (PMC10581408; doi:10.1249/MSS.0000000000003244)
Supplement: Supplementary file 2 [file msse-55-2014-s002.pdf]

Supplemental Digital Content 3

One participant (herein referred to as ppA) showed abnormal T_{gi} data at rest in the control condition, which strongly influenced the analysis of T_{gi} relative to baseline (ΔT_{gi}). Considering the uncertainty about the accuracy of this participant's data, we also performed the T_{gi} analyses without these data. The results are presented below, as well as the T_{gi} curves of the respective participant.

1-way repeated measures analyses, main effects

	**Test**	**Condition**
Peak T_{gi}, $n = 12$	ANOVA	F(1.9) = 5.6, p = **0.01**, $\eta^2_G = 0.05$
Peak T_{gi}, $n = 11$ (exclusion ppA)	ANOVA	F(2.5) = 10.2, p < **0.001** , $\eta^2_G = 0.08$
ΔT_{gi}, $n = 12$	ANOVA	F(2.0) = 1.4, p = 0.26, $\eta^2_G = 0.03$
ΔT_{gi}, $n = 11$ (exclusion ppA)	ANOVA	F(2.3) = 4.9, p = **0.02**, $\eta^2_G = 0.07$

T_{gi}, gastrointestinal temperature.

Pairwise comparisons for significant effects

	**COOL-UB**	**COOL-LB**	**CON**	**COOL-UB - CON**	**COOL-LB - CON**	**COOL-UB – COOL-LB**
Peak T_{gi} (°C), $n = 12$	38.0 ± 0.4	38.2 ± 0.4	38.3 ± 0.5	-0.2 (-0.4, -0.1) °C, t(11) = -4.0, p = **0.006**	-0.1 (-0.3, 0.1) °C, t(11) = -1.1, p = 0.88	-0.1 (-0.3,0.0) °C, t(11) = -2.2, p = 0.16
Peak T_{gi} (°C), $n = 11$ (ex. ppA)	38.1 ± 0.4	38.2 ± 0.4	38.4 ± 0.5	-0.3 (-0.4, -0.1) °C, t(10) = -4.8, p = **0.002**	-0.2 (-0.3, 0.0) °C, t(10) = -2.6, p = 0.08	-0.1 (-0.3, 0.0) °C, t(10) = -1.7, p = 0.34
ΔT_{gi} (°C), $n = 11$ (ex. ppA)	1.1 ± 0.4	1.2 ± 0.4	1.4 ± 0.4	-0.3 (-0.4, -0.1) °C, t(10) = -3.4, p = **0.02**	-0.2 (-0.4, 0.1) °C, t(10) = -1.7, p = 0.34	-0.1 (-0.3, 0.1) °C, t(10) = -1.2, p = 0.78

COOL-UB, upper-body cooling; COOL-LB, lower-body cooling; CON, control; T_{gi}, gastrointestinal temperature; Average data are presented as mean ± SD. Mean differences are presented as mean (95% CI).

T_{gi} curves for ppA

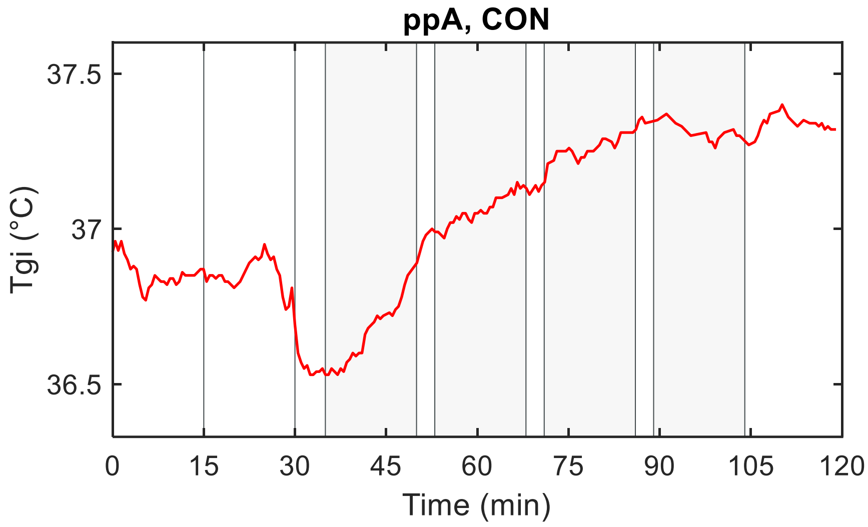

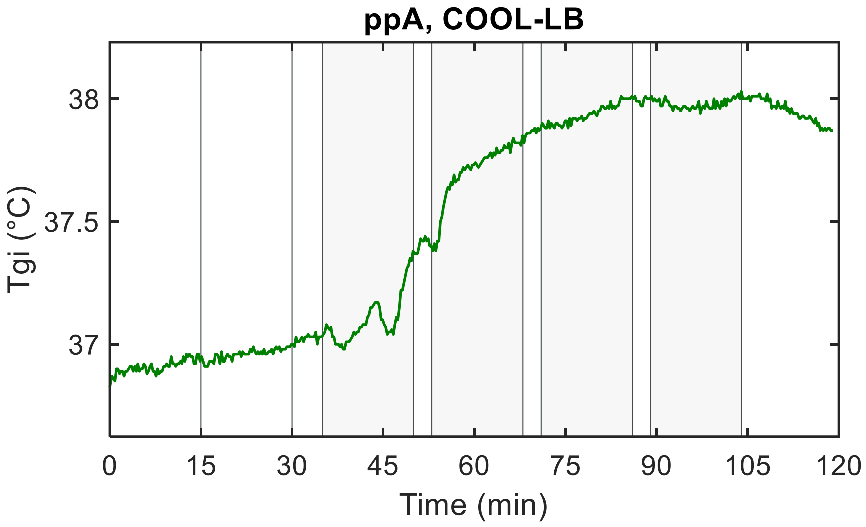

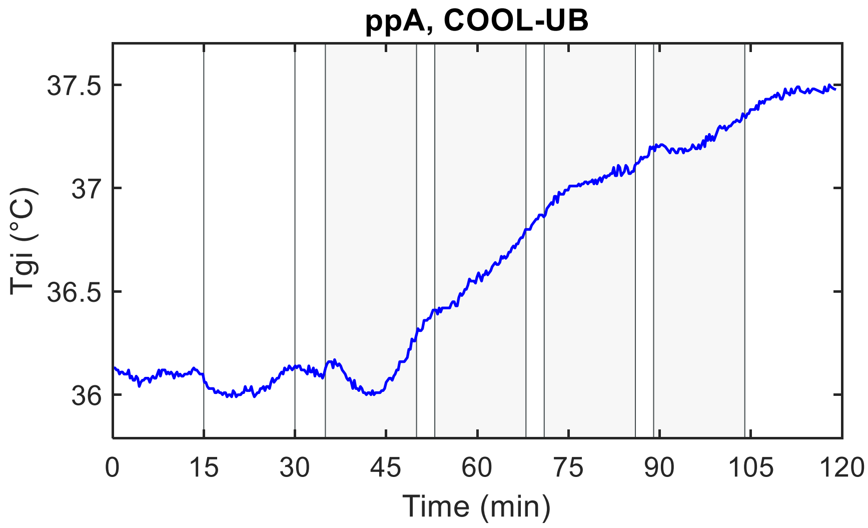

Comments:
Min 0-15: Rest (with min 10-15 = baseline measure).
Min 15-30: Rest with cooling.
Min 30-35: Warm-up at 20W.
Min 35-105: 4 exercise blocks of 15 min.

In CON, T_{gi} shows a sudden drop of ~0.3 °C just before exercise, for unclear reasons. We suspect it does not accurately represent a change in core temperature, and that it is an artefact (e.g., due to displacement of the capsule in the intestines).
