# Supplementary material for: Upper-Body versus Lower-Body Cooling in Individuals with Paraplegia during Arm-Crank Exercise in the Heat
Source: Med Sci Sports Exerc. 2023 Jun 30;55(11):2014–24. doi: 10.1249/MSS.0000000000003244 (PMC10581408; doi:10.1249/MSS.0000000000003244)
Supplement: Supplementary file 3 [file msse-55-2014-s003.docx]

**Supplemental Digital Content 2**

**2-way repeated measures ANOVAs**

**Main and interaction effects**

|  | **Condition** | **Time** | **Condition*blocks** |
| --- | --- | --- | --- |
| Tgi | F(1.8) = 4.3, *p* = **0.03**, η^2^_G_ = 0.07 | F(1.2) = 75.0, *p* **< 0.001**, η^2^_G_ = 0.48 | F(2.6) = 3.1, *p* = 0.051, η^2^_G_ < 0.01 |
| Heart rate | F(2.5) = 7.6, *p* = **0.004**, η^2^_G_ = 0.08 | F(1.5) = 34.3, *p* **< 0.001**, η^2^_G_ = 0.34 | F(1.7) = 0.6, *p* = 0.52, η^2^_G_ < 0.01 |
| Forehead Tsk | F(2.1) = 1.1, *p* = 0.36, η^2^_G_ = 0.03 | F(1.3) = 2.9, *p* = 0.11, η^2^_G_ = 0.05 | F(2.3) = 1.6, *p* = 0.22, η^2^_G_ = 0.03 |
| Upper-body Tsk | F(1.1) = 198, *p* **< 0.001**, η^2^_G_ = 0.9 | F(1.4) = 0.8, *p* = 0.44, η^2^_G_ < 0.01 | F(2.3) = 1.1, *p* = 0.35, η^2^_G_ < 0.01 |
| Lower-body Tsk | F(1.3) = 976, *p* **< 0.001**, η^2^_G_ = 0.9 | F(1.2) = 0.7, *p* = 0.44, η^2^_G_ < 0.01 | F(1.7) = 22, *p* **< 0.001**, η^2^_G_ = 0.07 |
| Forehead LSR | F(1.4) = 1.3, *p* = 0.30, η^2^_G_ < 0.01 | F(1.1) = 27.8, *p* **< 0.001**, η^2^_G_ = 0.19 | F(3.4) = 0.6, *p* = 0.66, η^2^_G_ < 0.01 |

Tgi, gastrointestinal temperature; Tsk, skin temperature; η^2^_G_, generalized eta squared.

**Pairwise comparisons significant main effects**

Because there was only a main effect, condition means were used for pairwise comparisons

|  | **COOL-UB** | **COOL-LB** | **CON** | **COOL-UB - CON** | **COOL-LB - CON** | **COOL-UB – COOL-LB** |
| --- | --- | --- | --- | --- | --- | --- |
| Tgi (°C) | 37.5 ± 0.4 | 37.7 ± 0.3 | 37.7 ± 0.3 | -0.2 (-0.3, -0.1) °C,  t(11) = -3.8, *p* = **0.009** | -0.02 (-0.2, 0.2) °C,  t(11) = -0.3, *p* = 1 | -0.2 (-0.4, 0.02) °C,  t(11) = -2.0, *p* = 0.21 |
| Heart rate (bpm) | 147 ± 9 | 149 ± 10 | 154 ± 10 | -7 (-11, -3) bpm,  t(10) = -3.7, *p* = **0.01** | -5 (-9, -1) bpm,  t(10) = -2.9, *p* = **0.049** | -2 (-6, 2) bpm,  t(10) = -1.0, *p* = 1 |
| UB Tsk (°C) | 29.0 ± 1.9 | 35.8 ± 0.4 | 35.7 ± 0.7 | -6.7 (-7.7, -5.8) °C,  t(10) = -15.7, *p* **< 0.001** | - 1. (-0.2, 0.4) °C,   t(10) = 0.6, *p* = 1 | -6.8 (-7.9, -5.7) °C, t(10) = -13.3, ***p* < 0.001** |

COOL-UB, upper-body cooling; COOL-LB, lower-body cooling; CON, control; Tgi, gastrointestinal temperature; Tsk, skin temperature; UB, upper body. Data are presented as mean ± SD.

**Pairwise comparisons significant interaction effects**

Simple main effects:

|  | **Exercise block** | **Condition** |
| --- | --- | --- |
| Tgi  *(tendency for interaction,*  *p = 0.051)* | 1 | F(2.0) = 2.1, p = 0.14, η^2^_G_ = 0.05 |
|  | 2 | F(1.7) = 2.6, *p* = 0.11, η^2^_G_ = 0.07 |
|  | 3 | F(1.5) = 4.8, *p* = **0.03**, η^2^_G_ = 0.08 |
|  | 4 | F(2.0) = 7.4, *p* = **0.004**, η^2^_G_ = 0.09 |
| LB Tsk | 1 | F(1.4) = 710, *p* **< 0.001**, η^2^_G_ = 0.9 |
|  | 2 | F(1.4) = 1061, *p* **< 0.001**, η^2^_G_ = 0.9 |
|  | 3 | F(1.4) = 852, *p* **< 0.001**, η^2^_G_ = 0.9 |
|  | 4 | F(1.4) = 632, *p* **< 0.001**, η^2^_G_ = 0.9 |

LB, lower body; Tsk, skin temperature; η^2^_G_, generalized eta squared.

**Tgi** (tendency for interaction, *p*=0.051): Pairwise comparisons among conditions, for each exercise block:

| **Block** | **COOL-UB** | **COOL-LB** | **CON** | **COOL-UB - CON** | **COOL-LB - CON** | **COOL-UB – COOL-LB** |
| --- | --- | --- | --- | --- | --- | --- |
| 1 | 37.0 ± 0.4 | 37.2 ± 0.2 | 37.2 ± 0.3 | -0.1 (-0.3, 0.0) °C,  t(11) = -1.5, *p* = 0.45 | 0 (-0.1, 0.2) °C,  t(11) = 0.65, *p* = 1.0 | -0.2 (-0.4, 0.0) °C,  t(11) = -1.7, *p* = 0.36 |
| 2 | 37.4 ± 0.3 | 37.6 ± 0.3 | 37.6 ± 0.3 | -0.2 (-0.3, 0.0) °C,  t(11) = -2.7, *p* = 0.058 | 0 (-0.2, 0.2) °C,  t(11) = 0.1, *p* = 1.0 | -0.2 (-0.4, 0.1) °C,  t(11) = -1.7, *p* = 0.38 |
| 3 | 37.7 ± 0.4 | 37.9 ± 0.3 | 37.9 ± 0.4 | -0.2 (-0.3, -0.1) °C,  t(11) = -4.7, *p* = **0.002** | -0.1 (-0.2, 0.1) °C,  t(11) = -0.63, *p* = 1.0 | -0.2 (-0.4, 0.0) °C,  t(11) = -2.0, *p* = 0.23 |
| 4 | 37.8 ± 0.4 | 38.0 ± 0.4 | 38.1 ± 0.5 | -0.3 (-0.4, -0.2) °C,  t(11) = -4.7, *p* = **0.002** | -0.1 (-0.3, 0.1) °C,  t(11) = -1.0, *p* = 0.96 | -0.2 (-0.4, 0.0) °C,  t(11) = -2.6, *p* = 0.08 |

COOL-UB, upper-body cooling; COOL-LB, lower-body cooling; CON, control. Data are presented as mean ± SD.

**Tsk,lb:** Pairwise comparisons among conditions, for each exercise block:

| **Block** | **COOL-UB** | **COOL-LB** | **CON** | **COOL-UB - CON** | **COOL-LB - CON** | **COOL-UB – COOL-LB** |
| --- | --- | --- | --- | --- | --- | --- |
| 1 | 32.1 ± 1.4 | 22.7 ± 1.4 | 32.4 ± 1.4 | -0.3 (-0.7, 0.1) °C,  t(10) = -1.7, *p* = 0.38 | -9.7 (-10.4, -8.9) °C,  t(10) = -28.3, *p* < **0.001** | 9.4 (8.6, 10.1) °C,  t(10) = 28.8, *p* < **0.001** |
| 2 | 32.4 ± 1.3 | 21.9 ± 1.7 | 32.6 ± 1.4 | -0.2 (-0.5, 0.2) °C,  t(10) = -1.2, *p* = 0.74 | -10.7 (-11.4, -10.0) °C,  t(10) = -35.2, *p* < **0.001** | 10.5 (9.8, 11.2) °C,  t(10) = 33.7, *p* **< 0.001** |
| 3 | 32.8 ± 1.2 | 21.5 ± 2.0 | 32.8 ± 1.4 | -0.1 (-0.5, 0.3) °C,  t(10) = -0.39, *p* = 1 | -11.3 (-12.1, -10.4) °C,  t(10) = -29.4, *p* **<** **0.001** | 11.2 (10.5, 12.0) °C,  t(10) = 32.9, *p* **< 0.001** |
| 4 | 33.0 ± 1.1 | 21.3 ± 2.2 | 33.1 ± 1.4 | -0.1 (-0.6, 0.4) °C,  t(10) = -0.3, *p* = 1 | -11.7 (-12.7, -10.7) °C,  t(10) = -25.5, *p* **< 0.001** | 11.7 (10.7, 12.6) °C,  t(10) = 28.1, *p* **< 0.001** |

COOL-UB, upper-body cooling; COOL-LB, lower-body cooling; CON, control. Data are presented as mean ± SD.

**1-way repeated measures analyses**

**Main effects**

|  | **Test** | **Condition** |
| --- | --- | --- |
| Peak Tgi | ANOVA | F(1.9) = 5.6, *p* = **0.01**, η^2^_G_ = 0.05 |
| Peak heart rate | ANOVA | F(2.5) = 2.6, p = 0.10, η^2^_G_ = 0.06 |
| WBSL | Friedman | χ^2^(2) = 9.0, *p* = **0.01**, W = 0.45 |
| Median whole-body thermal sensation | Friedman | χ^2^(2) = 5.4, *p* = 0.07, W = 0.23 |
| Median upper-body thermal sensation | Friedman | χ^2^(2) = 17.2, *p* **< 0.001**, W = 0.72 |
| Median lower-body thermal sensation | Friedman | χ^2^(2) = 7.4, *p* = **0.02**, W = 0.31 |
| Median thermal comfort | Friedman | χ^2^(2) = 7.5, *p* = **0.02**, W = 0.31 |
| Median RPE | Friedman | χ^2^(2) = 0.05, *p* = 1, W = 0.002 |

Tgi, gastrointestinal temperature; WBSL, whole-body sweat loss; η^2^_G_, generalized eta squared; W, Kendall’s W.

**Pairwise comparisons significant effects**

|  | **COOL-UB** | **COOL-LB** | **CON** | **COOL-UB - CON** | **COOL-LB - CON** | **COOL-UB – COOL-LB** |
| --- | --- | --- | --- | --- | --- | --- |
| Peak Tgi (°C) | 38.0 ± 0.4 | 38.2 ± 0.4 | 38.3 ± 0.5 | -0.2 (-0.4, -0.1) °C,  t(11) = -4.0, *p* = **0.006** | -0.1 (-0.3, 0.1) °C,  t(11) = -1.1, *p* = 0.88 | -0.1 (-0.3,0.0) °C,  t(11) = -2.2, *p* = 0.16 |
| WBSL (kg) | 0.87  [0.76–0.99] | 0.85  [0.72–0.92] | 0.98  [0.90–1.0] | -0.10 [-0.21–-0.04] kg,  Z = 9, *p* = 0.37 | -0.10 [-0.26–-0.04] kg,  Z = 9, *p* = 0.20 | 0.03 [-0.07–0.25] kg,  Z = 34, *p* = 1 |
| Upper-body TS (A.U.) | 0.5  [-0.5–1.1] | 2.0  [1.0–2.3] | 2.0  [1.9–3.0] | -1.8 [-2.6–-1.0],  Z = 0, *p* = **0.01** | -0.75 [-1.0–0.0],  Z = 7, *p* = 0.20 | -1.3 [-2.0–0.5],  Z = 0, *p* = **0.02** |
| Lower-body TS (A.U.) | 0.0  [0.0, 0.0] | 0.0  [-0.25, 0.0] | 0.0  [0.0, 0.3] | 0.0 [-0.1–0.0],  Z = 4, *p* = 1 | 0.0 [-0.6–0.0],  Z = 0, *p* = 0.17 | 0.0 [0.0–1.0],  Z = 10, *p* = 0.27 |
| Thermal comfort (A.U.) | 1.0  [0.5–1.5] | 1.0  [1.0–1.5] | 1.5  [1.0–2.0] | -0.5 [-1.0–-0.4],  Z = 3.5, *p* = **0.04** | -0.5 [-0.5–0.0],  Z = 11.5, *p* = 0.59 | -0.3 [-1.0–0.13],  Z = 9, *p* = 0.34 |

COOL-UB, upper-body cooling; COOL-LB, lower-body cooling; CON, control; Tgi, gastrointestinal temperature; WBSL, whole-body sweat loss; TS, thermal sensation. Average values are presented as mean ± SD or median [first quartile – third quartile]. Mean differences are presented as mean (95% CI) or median [first quartile – third quartile].
